# Single Polysaccharide Dissolvable Microneedles for Painless Local Anesthesia: Fabrication, Characterization, and *In Vitro* Neuronal Imaging

**DOI:** 10.1021/acsbiomaterials.5c00968

**Published:** 2025-08-01

**Authors:** João M. S. P. Leite, Ana C. Q. Silva, Ana Jesus, Bruna C. da Cruz, Sandra I. Vieira, Patrícia Dias-Pereira, Paulo C. Costa, Isabel F. Almeida, Inês Correia-Sá, Armando J. D. Silvestre, Bruno M. Neves, Carla Vilela, Carmen S. R. Freire

**Affiliations:** † CICECO − Aveiro Institute of Materials, Department of Chemistry, 56062 University of Aveiro, Aveiro 3810-193, Portugal; ‡ iBiMED − Institute of Biomedicine (iBiMED), Department of Medical Sciences, University of Aveiro, Aveiro 3810-193, Portugal; § ICBAS − Institute of Biomedical Sciences Abel Salazar, University of Porto, Porto 4050-313, Portugal; ∥ Associate Laboratory i4HB - Institute for Health and Bioeconomy, Faculty of Pharmacy, 131671 University of Porto, Porto 4050-313, Portugal; ⊥ UCIBIO − Applied Molecular Biosciences Unit, Department of Drug Sciences, Faculty of Pharmacy, University of Porto, Porto 4050-313, Portugal; # UCIBIO - Applied Molecular Biosciences Unit, MedTech−Laboratory of Pharmaceutical Technology, Faculty of Pharmacy, University of Porto, Porto 4050-313, Portugal; ∇ Department of Plastic, Reconstructive and Aesthetic Surgery, ULS São João, Porto 4200-319, Portugal; ○ LAQV/REQUIMTE - Associated Laboratory for Green Chemistry, University of Porto, Porto 4050-313, Portugal

**Keywords:** microneedles, pullulan, lidocaine, local anesthesia, drug delivery

## Abstract

In recent years, microneedles (MNs) have shown high potential as drug delivery devices capable of administering different drugs in a simple, fast, and minimally invasive manner. Their ability to pierce the stratum corneum barrier heavily outweighs the inconveniences posed by conventional administration methods such as hypodermal injections, creams, or ointments. In this work, high-performance, single-component polysaccharide-based microneedles, *viz*. pullulan MNs, were produced by micromolding, aimed for transdermal delivery of lidocaine. These dissolvable MNs were able to sustain forces up to 1.5 N per needle without breakage. Their ability to overcome the stratum corneum was validated using excised human abdominal skin, with the MNs successfully reaching the dermis. The MN tips completely dissolved after 10 min in an agarose gel skin model and porcine ear skin, with 26 and 32% of lidocaine being retained in the patch after 10 min of insertion in the agarose gel and porcine skin, respectively. Cytocompatibility against multiple cell lines (HaCaT, 3T3, and RAW 264.7) was demonstrated by the resazurin metabolic assay, with over 80% cell viability in all cases. Time-lapse fluorescence microscopy confirmed that the activity of the MN-released lidocaine on sensory neurons was comparable to that of a pure lidocaine solution at a similar concentration. In sum, given its low onset time (<10 min), swift dissolution, and effective drug release, this pullulan-based system constitutes a simple, safe, and efficient delivery device, contrasting conventional methods of local anesthetic administration.

## Graphical abstract

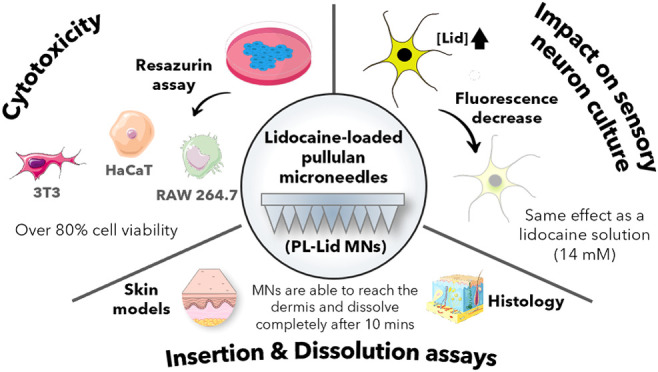

## 1 Introduction

The human body’s leading alarm mechanism against injury is none other than the experience of pain. This feeling, either chronic or acute, is a source of discomfort and mental distress that hinders the normal performance of basic activities.^1^ Pain management is an important healthcare issue, crucial for the success of several clinical interventions (*e*.*g*., surgeries), without which the treatment of certain health conditions would be very difficult.^2^ In multiple situations, pain control can be achieved using local anesthetics. These drugs are responsible for blocking the transmission of nerve impulses to the brain, eliminating pain in a specific area of the body, without the loss of consciousness.^3^ Lidocaine is the most widely used anesthetic, being well-established in clinical practice due to its effectiveness, few side effects, great water solubility, fast onset, and low risk of anaphylaxis.^1,4^ The effect of lidocaine has been well investigated *in vitro* and *in vivo*, and its main mechanism of action is through blockage of voltage-gated sodium channels present at the neuronal cell membrane.^5,6^

Currently, the main administration routes for local anesthesia are by direct injection into the tissue and transdermal delivery.^1^ However, on the one hand, hypodermal injections require trained personnel for their administration and are often haunted by trypanophobia, which can lead to rejection of the procedure by the patient.^3^ On the other hand, transdermal administration, which often relies on the use of creams, ointments, or patches, is favored by having fewer side effects, high patient compliance, and minimal discomfort.^1,7,8^ However, the skin’s main barrier, the stratum corneum (SC), significantly disturbs passive drug diffusion, which consequently leads to a slow diffusion process until the drug reaches the peripheral nerves and produces the desired effect.^3,9^ For instance, transdermal lidocaine patches for treating peripheral neuropathic pain often require patch-wear times up to 1 h, and some randomized controlled trials have shown that the pain-reducing effect was only achieved 4 h after patch application.^10^ The same trend was observed with patches aimed for chronic pain treatment, requiring up to 4 patches a day, culminating in a total daily wear time of 12 to 18 h.^10^ Importantly, long patch-wear durations have been documented to cause skin rash, erythema, and obvious discomfort.^10^ Therefore, an effective skin drug delivery system should present a high ability to overcome the SC and to quickly reach the viable epidermis or dermis.^7^

Microneedles (MNs) are minimally invasive microscale systems highlighted for their ability to penetrate the SC, with proven enhanced delivery of several drugs.^11,12^ The fact that they can be self-administered, without requiring the intervention of trained professionals, is another advantage of these systems.^13^ Among the several classes of MNs, dissolvable microneedles (dMNs) have gained considerable interest in the field, owing to their facile fabrication, rapid onset of action, and high drug loading capacity.^14,15^ Upon skin insertion, dMNs deliver their payload through dissolution of the microneedle tips, facilitating diffusion directly into the dermis.^14^ The choice of the material for dMN fabrication is thus crucial, and water-soluble materials are obviously required for this application.^14,16^ Water-soluble synthetic or natural polymers (alias biopolymers) can be used, but natural polymers have superior biocompatibility, biodegradability, and ease of chemical modification over synthetic ones.^16,17^ Although several dMNs have been developed for local anesthesia,^18–21^ only a few studies have exploited biopolymers for their fabrication. Most of these studies focused on the use of hyaluronic acid,^19,22–24^ carboxymethyl cellulose,^3,25^ and chondroitin sulfate,^26^ frequently requiring a combination of two or more biopolymers to achieve proper mechanical performance. This gap paves the way for the exploration of other biopolymeric MNs as vehicles for the transdermal administration of anesthetics.^27^

Pullulan is an exopolysaccharide produced by the aerobic fermentation of a yeast-like fungus (*Aureobasidium pullulans*). It is known for being edible, water-soluble, odorless, tasteless, nonmutagenic, biocompatible, and biodegradable.^28^ Structurally, pullulan is a linear unbranched biopolymer comprising α-(1,4)-linked maltotriose repeating units interconnected through α-(1,6) glycosidic bonds.^28^ This distinct linkage pattern is responsible for its outstanding film-forming capabilities and exceptional mechanical strength. Such features have made pullulan a viable asset in the development of advanced materials for various biomedical applications, such as hydrogels for vaccine delivery,^29^ films and coatings with excellent barrier properties for packaging applications,^30^ scaffolds for bone tissue engineering,^31^ and drug delivery systems.^28,32^ Pullulan dMNs have also been prepared for wound healing,^33^ vaccine administration,^34^ and delivery of several drugs such as insulin and moxifloxacin.^35,36^ However, to the best of our knowledge, the potential of pullulan for the fabrication of dissolvable MNs for local anesthesia has not yet been studied. Herein, dissolvable lidocaine-loaded pullulan microneedles were produced through micromolding for local anesthesia application. This system was thoroughly characterized in terms of its mechanical properties, skin insertion potential, safety, and also regarding the activity of the released drug, overall showcasing a high-performing device.

## 2 Materials and Methods

### 2.1 Materials and Reagents

Pullulan (98%, molecular weight 272 kDa) was acquired from B&K Technology Group (China). Lidocaine hydrochloride monohydrate (Lid) (99.5%) was purchased from Sigma-Aldrich (Sintra, Portugal). Ultrapure water (Type 1, 18.2 MΩ·cm resistivity (25 °C) at 0.5 L min^–1^) was obtained by a Simplicity Water Purification System (Merck, Darmstadt, Germany). Phosphate-buffered saline (PBS, pH 7.4) was supplied from Gibco (Life Technologies, Carlsbad, CA). Dulbecco’s modified Eagle’s medium (DMEM), DMEM-F12 Glutamax, fetal bovine serum (FBS), and penicillin–streptomycin (10,000 U mL^–1^) were purchased from Gibco (Paisley, UK). Resazurin sodium salt (powder, BioReagent, suitable for cell culture) was acquired from Sigma-Aldrich (St. Louis, Missouri). Molecular probe FM1–43FX was acquired from Invitrogen (Massachusetts).

### 2.2 Fabrication of the MN Patches

MN patches were produced by micromolding using polydimethylsiloxane female master molds (15 × 15 pyramidal needles, 64 mm^2^) with a needle height of 550 μm, base of 200 μm, and pitch of 500 μm (Micropoint Technologies Pte Ltd., Singapore). Two different types of patches were produced, namely, patches comprising only pullulan (PL MNs) and pullulan patches loaded with lidocaine (PL-Lid MNs). Briefly, a 20% (w/v) pullulan solution was prepared by dissolving 2.0 g of pullulan in ultrapure water (10 mL) at 40 °C under constant stirring. The resulting solution was sealed with Parafilm M and set to rest overnight to remove potential air bubbles caused by the stirring. Subsequent storage of this solution is held at 4 °C. Then, the process involves careful placing of the solution into the molds with a spatula, which are afterward sealed and centrifuged at 6000 rpm for 5 min to allow the solution to fill the mold cavities. This centrifugation step was repeated three times to ensure that all cavities are properly filled and air bubbles are removed. Then, more pullulan solution was added to the mold until a total mass of 110 mg was achieved, and the mold was left to dry overnight in an oven at 28 °C. Finally, the MN patches were carefully pulled off from the molds and stored in a desiccator. PL-Lid MNs were fabricated following the same methodology, but adding 200 mg of lidocaine into 10 mL of ultrapure water prior to the addition of pullulan and its subsequent dissolution, in order to achieve a lidocaine amount of approximately 2 mg per patch, which is required.

### 2.3 Morphological Characterization

PL and PL-Lid MNs were analyzed by using a stereomicroscope (Nikon SMZ18, Tokyo, Japan) coupled with an SRH Plan Apo 2 camera (Tokyo, Japan). The obtained images were processed using NIS Elements Imaging Software, which was also employed to perform height measurements of the needles. Scanning electron microscopy (SEM) images were acquired using a high-voltage microscope (Hitachi SU 70, Tokyo, Japan) operating at 15 kV. Previously, the MN patches were placed in an aluminum support and coated with a carbon film (EMITECH K950, Laughton, UK).

### 2.4 Fourier Transform Infrared-Attenuated Total Reflection (FTIR-ATR) Spectroscopy

FTIR-ATR spectra were acquired on a PerkinElmer FTIR System Spectrum BX spectrophotometer (PerkinElmer Inc.) equipped with a single horizontal Golden Gate ATR cell. Acquisition was conducted with 32 scans over the range of 4000–600 cm^–1^, with a resolution of 2 cm^–1^.

### 2.5 Thermogravimetric Analysis (TGA)

TGA was performed with a SETSYS Setaram TGA analyzer (SETARAM Instrumentation, France) equipped with a platinum cell. Samples were heated in an inert atmosphere (nitrogen flow) from room temperature to 800 °C at a constant rate of 10 °C min^–1^.

### 2.6 Mechanical Characterization

To evaluate the mechanical performance of the produced MN patches, axial compression tests were performed by using a TA.XT2 texture analyzer (Stable Micro Systems Ltd., Haslemere, UK) equipped with a flat-ended P/2 (2 mm diameter) cylinder probe. In order to replicate the force undertaken by the needles upon skin insertion, an axial force was applied at a constant speed of 0.01 mm s^–1^. Data acquisition began at the instant the probe touches the tip of the needles, recording the applied force as a function of displacement for nine patches of each type (*n* = 9).

### 2.7 Insertion in a Parafilm M model

To assess the penetration potential of the MN patches, preliminary insertion tests were carried out using the TA.XT2 texture analyzer (Stable Micro Systems Ltd., Haslemere, UK) equipped with a P/6 (6 mm diameter) probe. The MN patches were pressed against a skin film model consisting of 8 stacked layers of Parafilm M (Bemis Company Inc., Soignies, Belgium) with a constant force of 40 N over 30 s.^37^ Afterward, the MNs were carefully removed from the skin model film, and each Parafilm M layer was observed under an optical microscope (Olympus BX51, Olympus Corporation, Tokyo, Japan), counting the number of insertions per layer and calculating the percentages regarding the number of perforations in the first layer (100% insertion) (*n* = 6). Photographs of each layer were also obtained to observe the MN insertion marks.

### 2.8 *In Vitro* Insertion in Excised Human Skin

To confirm their potential for skin penetration, PL-Lid MNs were also inserted into excised human abdominal skin tissue recovered from a female donor that underwent an abdominoplasty performed at Centro Hospitalar e Universitário S. João (CHUSJ), Porto, Portugal. The study was conducted in accordance with the Declaration of Helsinki and approved by the Ethics Committee of CHUSJ (Protocol 61–15, approved on May 13, 2015). Skin samples were stored in a freezer at −20 °C prior to use. A biopsy punch (25 mm in diameter) was used to cut the skin sample into circles, which were then hydrated with PBS-embedded cotton for 1 h inside a sealed Petri dish. Afterward, the skin circles were fixed onto a solid platform with the aid of hypodermic needles. Insertion was carried out using the texture analyzer (Texture Technologies, Hamilton, MA), pressing the MN patches against the skin using a constant force of 40 N for 30 s. After insertion, the MN patch was removed, and China ink was spread through the surface of the skin insertion area in order to reveal the insertion marks left by the MNs. For histological examination, samples were immersed in Bouin’s solution and fixed in 10% formalin, followed by dehydration and fixation in paraffin wax. Serial sections of 7 μm were cut using a Rotary 3006 EM automated microtome, stained with hematoxylin and eosin, and observed under a microscope (Nikon Eclipse E600, Tokyo, Japan).

### 2.9 *In Vitro* Drug Release in PBS

The release profile of lidocaine in solution was investigated by dissolving PL-Lid MNs (*n* = 3, weighed beforehand) in a vial containing 3 mL of PBS at 37 °C under constant stirring at 200 rpm. Aliquots of 300 μL of the solution were collected at predetermined time points and replaced with the same volume of preheated PBS to maintain a constant volume and temperature. The amount of lidocaine released to the medium was quantified by ultraviolet–visible (UV–vis) absorption spectroscopy (Thermo Scientific Evolution 600, Thermo Fisher Scientific, Waltham MA) at λ = 263 nm using a pre-established calibration curve in the range of 0.01–0.11 mg mL^–1^ (*y* = 1.7081*x* + 0.0149; *R* ^2^ = 0.9939) and expressed as a cumulative release percentage over time with regard to the maximum lidocaine amount in each patch (10% of the mass of the patch).

### 2.10 *In Vitro* Dissolution Tests in Agarose Gel and Porcine Skin Models

To evaluate the dissolution profile of the PL-Lid MNs, two separate assays were performed, one using an agarose hydrogel (1.4% w/v) skin model known to replicate the viscoelastic properties of the skin,^38,39^ and the other using porcine ear skin.^40^ For the former, a single layer of Parafilm M was stretched and fixed on top of the hydrogel to mimic the resistance promoted by the SC upon insertion in the skin and also to allow only the needle projections to come into direct contact with the hydrogel. For the latter, porcine ear skin was kindly provided by a local butcher, carefully cut into small sections, and removed of excess fat. The skin was stretched and hydrated for 1 h with PBS-embedded cotton inside a sealed Petri dish, and excess PBS on the skin was removed with a dry tissue prior to MN insertion. In both assays, insertion of the patches was performed using a spring applicator (Micropoint Technologies Pte Ltd., Singapore) to ensure a constant insertion force between all of the tested patches (*n* = 6). At determined time points, the patches were photographed using an iPhone 11 Pro Max (2× amplification mode) immobilized with a phone support stand.

Moreover, patches were removed from both the hydrogel and porcine skin after 1, 2, 5, and 10 min of being inserted to quantify the amount of lidocaine retained in the patch. This was done by dissolving the leftover patch in 3 mL of PBS at 37 °C and then measuring the absorbance of the resulting solution through UV–vis spectroscopy (λ = 263 nm) to determine lidocaine concentration based on the aforementioned calibration curve. The percentage of retention is then calculated with respect to the previously weighted mass of each patch used. Micrographs of the needles before and after 5 min of insertion into agarose gel were taken by using a Nikon Eclipse E600 microscope (Tokyo, Japan).

### 2.11 In Vitro Cytotoxicity and Bioactivity Assays

For cell viability assays, the human keratinocyte cell line HaCaT (DKFZ, Heidelberg, Germany), the 3T3 mouse fibroblasts (ATCC N°. CRL-1658, Manassas), and the RAW 264.7 mouse macrophages (ATTC N°.TIB-71) were cultured in DMEM supplemented with 4 mM glutamine, 10% heat inactivated FBS, and 100 U mL^–1^ penicillin/streptomycin. These cells were used after reaching 70–80% confluence. For the bioactivity assays in neuron cell cultures, the F11 cell line (ECACC 08062601) was used. This is a somatic cell hybrid of a rat embryonic dorsal root ganglion (DRG) and mouse neuroblastoma cell line N18TG2. F11 cells were maintained in DMEM-F12 Glutamax supplemented with 10% heat inactivated FBS and 100 U mL^–1^ penicillin/streptomycin. To induce F11 differentiation, cells were seeded at 6 × 10^3^ cells/cm^2^ in poly-l-lysine (PLL) precoated plates, incubated for 2 days in DMEM-F12 Glutamax supplemented with 10 μM Forskolin (Sigma-Aldrich) and 0.5% FBS, and for further 2 days in the same medium but with serum reduced to 0.1% FBS. All of the cell lines were maintained at 37 °C in a humidified atmosphere of 95% air and 5% CO_2_, routinely monitored by microscope observation, and tested for mycoplasma contamination.

The MN patches used in these studies were previously sterilized by UV exposure (3 cycles of 30 min each) inside a flow chamber.

### 2.12 Cell Viability Assays

The effects of the produced MN patches on the viability/metabolic activity of macrophages, keratinocytes, and fibroblasts were assessed by the resazurin assay.^41^ Briefly, 0.5 × 10^6^ Raw 264.7, 0.1 × 10^6^ HaCaT, or 0.1 × 10^6^ 3T3 cells were plated in 1 mL of DMEM per well of a 12-well plate and allowed to stabilize overnight. Then, MN patches were put into contact with cell cultures during 24 h by means of Transwell inserts with 1 μm transparent polyester (cellQART, SABEU GmbH & Co. KG, Germany)). Resazurin was added to cells in a final concentration of 50 μM during the last 2 h of incubation, when 200 μL were transferred from each system to a 96-well plate. The absorbance of resorufin (the product of the resazurin reduction) was measured at 570 and 600 nm in a Tecan infinite M200 spectrophotometer (Tecan Group, Switzerland). The obtained data are the average of three biological independent experiments for each condition, and the results are expressed as the average cell viability ± standard deviation (SD).

### 2.13 Bioactivity Assays with Sensory Neurons

Lidocaine activates TRPA1 and TRPV1 channels, promoting fast endocytosis and compensatory exocytosis in neurons, while inhibiting their sodium channels.^42–44^ The activity of lidocaine on F11 sensory neurons was indirectly assessed through the quantification of the FM1–43FX fluorescence probe, an indicator of neuronal endocytosis and exocytosis, using two assays. In the first assay, cells were fixed after 5 min of exposure to a lidocaine solution or PL-Lid MNs. Briefly, F11 cells differentiated in 24-well plates were incubated on ice for 15 min prior to a 2 min staining with a cold 5 μg mL^–1^ solution of FM1–43FX in PBS. After washing the unbound probe with PBS, cells were incubated for 5 min at 37 °C with 7 mM or 14 mM lidocaine (“Lid”), with a solution of the content of microneedles dissolved in PBS, yielding approximately 14 mM lidocaine (“PL-Lid MN”), or with PBS only. Cells were immediately fixed with 4% paraformaldehyde, and fluorescence micrographs were acquired using an Olympus IX81 microscope (Tokyo, Japan). In the second assay, cells were loaded with 5 μg mL^–1^ solution of FM1–43FX in PBS and monitored by real-time live cell imaging after the addition of (i) a lidocaine solution with a concentration of 14 mM or (ii) PL-Lid MN dissolved in PBS; these agents were added at approximately 1–2 min after starting image acquisition. Control conditions corresponded to the basal decay of fluorescence (no lidocaine added). Fluorescence micrographs were acquired (ZEISS LSM 880 with Airyscan) at every second, in a total of 5–10 min of imaging per condition. In-house made Ca^2+^-free PBS (136.9 mM NaCl, 2.7 mM KCl, 9.1 mM Na_4_P_2_O_7_, and 1.8 mM KH_2_PO_4_) was used in both assays.

### 2.14 Image Analysis Using ImageJ

The micrographs of the neuronal cultures were processed and quantized using Fiji (ImageJ) software, namely, through its functionalities, to measure area, mean gray value, and integrated density. For fluorescence micrographs obtained in an Olympus IX81, mean fluorescence intensity (au) was determined in a region of interest (ROI) comprising all cell bodies and neurites of each image. ROI creation was achieved using trainable WEKA segmentation after mild image adjustments (*e*.*g*., thresholding) and with background intensity subtraction. For image stacks acquired in real-time live cell imaging, two separate measurements were performed for each image, namely, measurement of overall integrated density and ROI integrated density. This was done to verify the accuracy of the ROI through comparison with the overall measurements.

### 2.15 Statistical Analysis

Results are presented as mean values ± standard deviation (*n* ≥ 3). For *in vitro* cell culture assays and fluorescence intensity measurements, analysis of variance (One-way ANOVA) was performed to determine the statistical significance of the data established at *p* < 0.05. All aforementioned statistical tests were computed using GraphPad Prism software, version 8.0.1 (GraphPad Software Inc., San Diego, CA).

## 3 Results and Discussion

This work reports the fabrication of dissolvable lidocaine-loaded pullulan microneedle patches (PL-Lid MNs) by micromolding aimed at painless local anesthesia, as depicted in Figure 1. A pullulan solution at a concentration of 20% (w/v) was chosen to produce MNs with proper mechanical properties and sharp tips.^36,45^ To achieve the desired anesthetic effect, it is suggested that a lidocaine amount of approximately 2 mg per patch is required.^10,21,23,46^ Thus, a formulation was devised to produce PL MN patches with 10% (w/w) lidocaine for a total mass of approximately 20 mg, resulting in 2 mg of lidocaine upon full dissolution of the MNs. This MN system was developed to enable easy self-application with a fast onset time and was thoroughly characterized in terms of morphology, mechanical properties, insertion capabilities, drug release profile, biological safety against multiple cell lines (HaCaT, 3T3, and Raw 264.7), and impact of released lidocaine against DRG neuron cell line.

**Figure 1:**
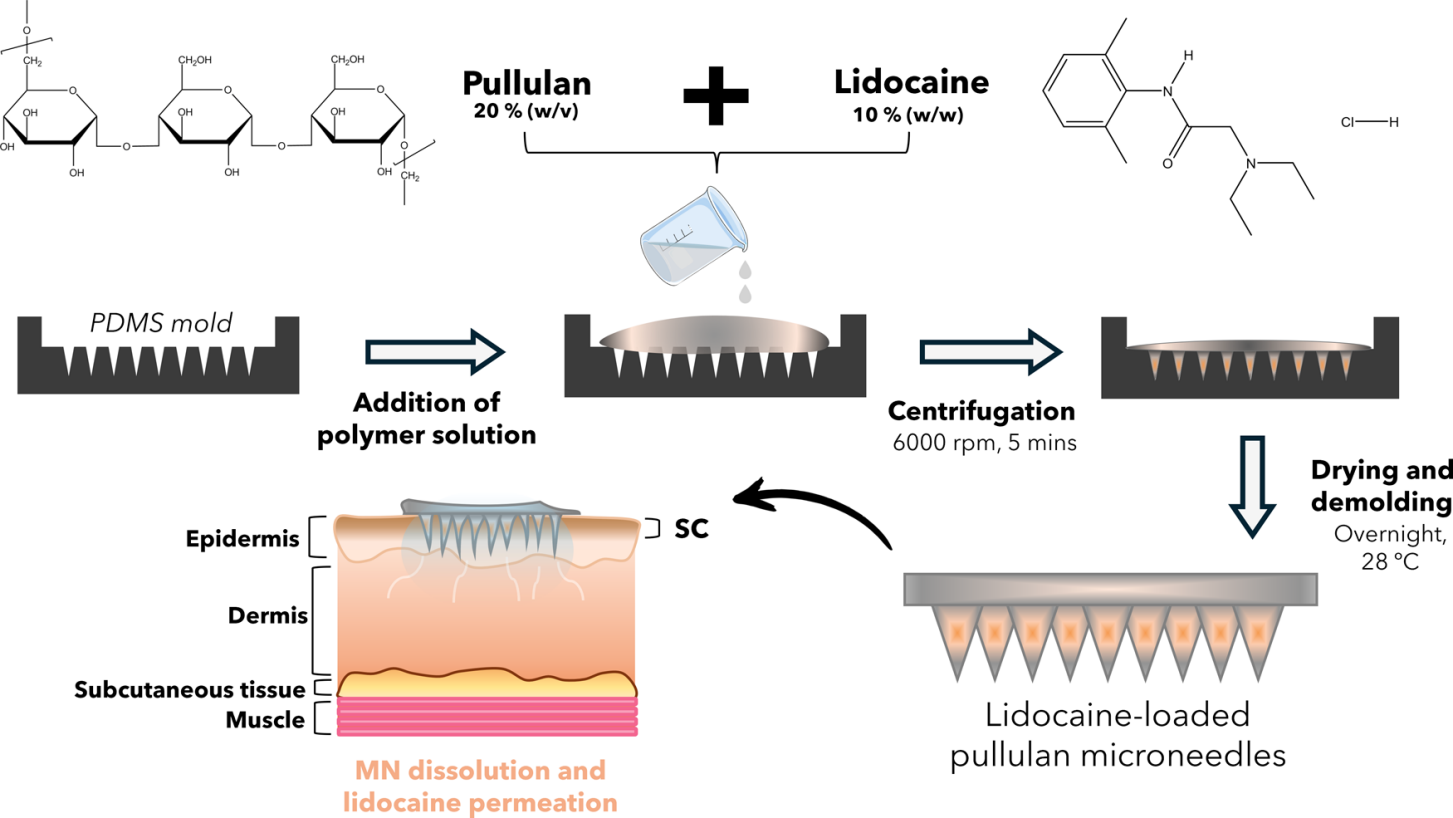
Schematic representation of the PL-Lid MN patch fabrication process and its applicability for local anesthesia.

### 3.1 Morphological Characterization

Optical and SEM micrographs of the patches are provided in Figure 2A,B, respectively. As clearly visible in the whole-patch images (Figure 2A), both PL MNs and PL-Lid MNs showcase a complete replication of the mold with all of the 225 needle projections. Optical micrographs reveal well-defined, evenly spaced pyramidal microneedles with sharp tips, and both types of MN patches display visually identical features, namely, the typical off-white coloration and transparency characteristic of pullulan.^47,48^ Therefore, the incorporation of lidocaine in the pullulan patches does not change their aspect, as they maintain their shape, color, and transparency without the formation of drug aggregates. This is a result of the complete dissolution of pullulan and lidocaine and the retention of this homogeneity during the fabrication process and particularly during the drying stage.

**Figure 2:**
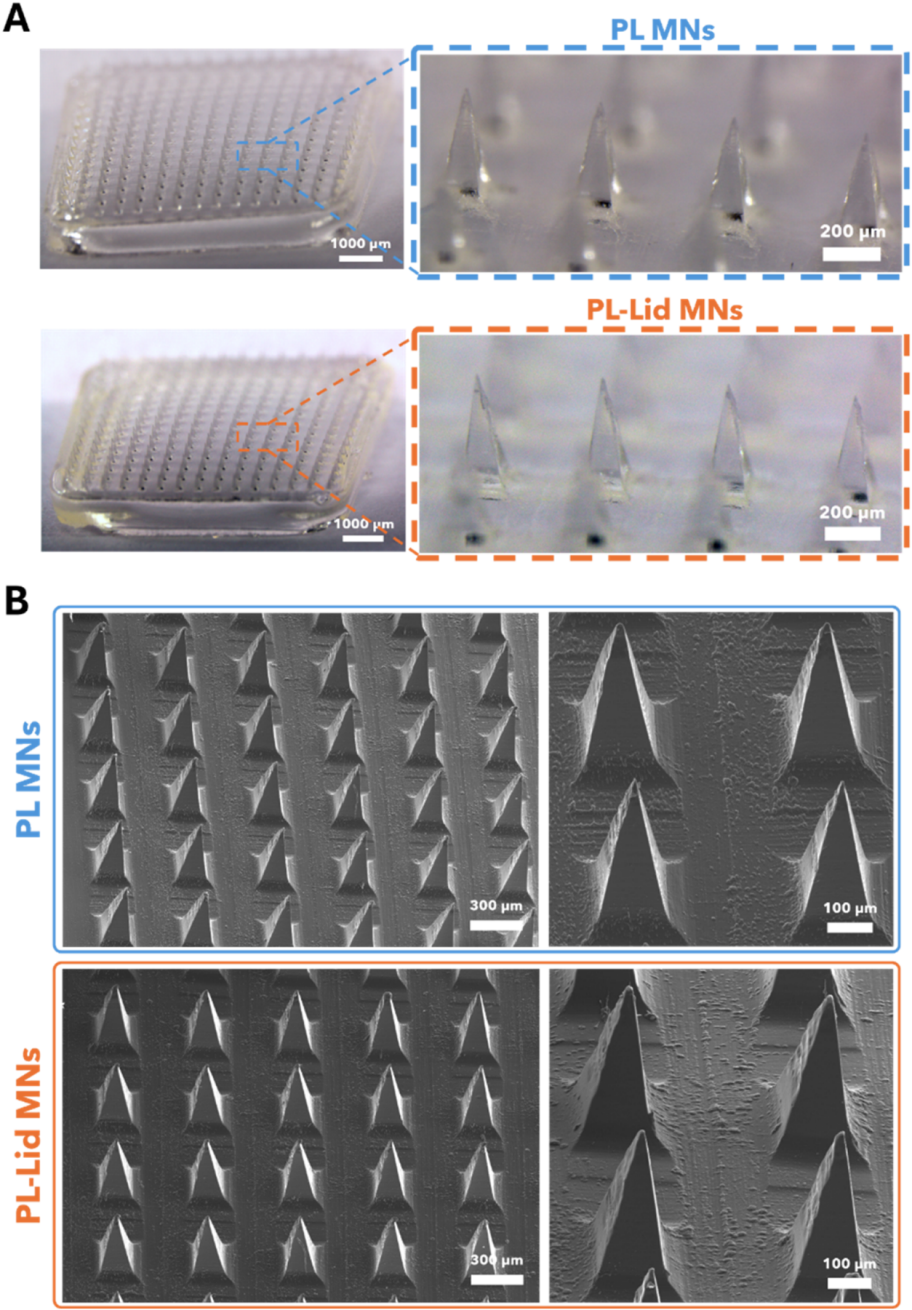
Optical (A) and SEM (B) micrographs of PL MNs and PL-Lid MNs patches.

An average needle height of 473 ± 21 and 485 ± 17 μm (*n* = 30) was measured for PL and PL-Lid MNs, respectively. A slight reduction in the needle’s height is observed comparatively to the mold’s dimensions, which was expected, since the drying process causes some shrinkage due to water evaporation.^18,49^ According to literature, pyramidal MNs tend to maintain over 75% of the mold’s original height;^7^ herein, the pyramidal PL and PL-Lid MNs kept over 86 and 88%, respectively, of the mold’s original height, which might be attributed to the selected biopolymer, its molecular weight, and concentration of the pristine aqueous solution. Similar findings were reported in other studies dealing with PL MNs for the administration of insulin^36,45^ and different model compounds such as methylene blue and fluorescein sodium,^45^ as well as regarding other biopolymeric MNs for lidocaine administration, for example, lidocaine-loaded hyaluronic acid MNs.^19^ Most importantly, the obtained needle height is deemed high enough to overcome the thickness of the human SC (50–100 μm), while being short enough to minimize the risk of pain upon insertion.^18,50,51^

The SEM micrographs (Figure 2B) provide a more in-depth view of the microneedle projections, evidencing a relatively smooth surface across all pyramidal faces without the presence of any cracks and bubbles and corroborating the absence of lidocaine aggregates. Furthermore, these images showcase uniformity across all needles of the patch with the absence of any broken tips, validating the integrity of the patch for skin insertion.

### 3.2 Fourier Transform Infrared-Attenuated Total Reflection (FTIR-ATR) Spectroscopy

FTIR-ATR spectroscopic analysis was carried out to verify the establishment of possible drug-polymer interactions during the MN fabrication process, with the obtained spectra shown in Figure 3A. The spectra of PL and PL MNs present the typical vibrations of a polysaccharide, namely, the O–H stretching vibration (ν_OH_) around 3300 cm^–1^ and C–H (ν_CH_) and H–C–H (ν_CH2_) stretching vibrations at 2929 and 1240–1460 cm^–1^, respectively.^36,52^ The H–O–H bending vibration (δ_H2O_) characteristic of adsorbed water is visible at 1650 cm^–1^. Between 1150 and 1070 cm^–1^, the band typical of the bending vibration of C–O–C glycosidic bridges (δ_COC_) is observed.^36,52^ The absence of noticeable differences between the spectra of PL and PL MNs indicates, as expected, that the dissolution of this polymer and the micromolding procedure do not compromise its structural features.

**Figure 3:**
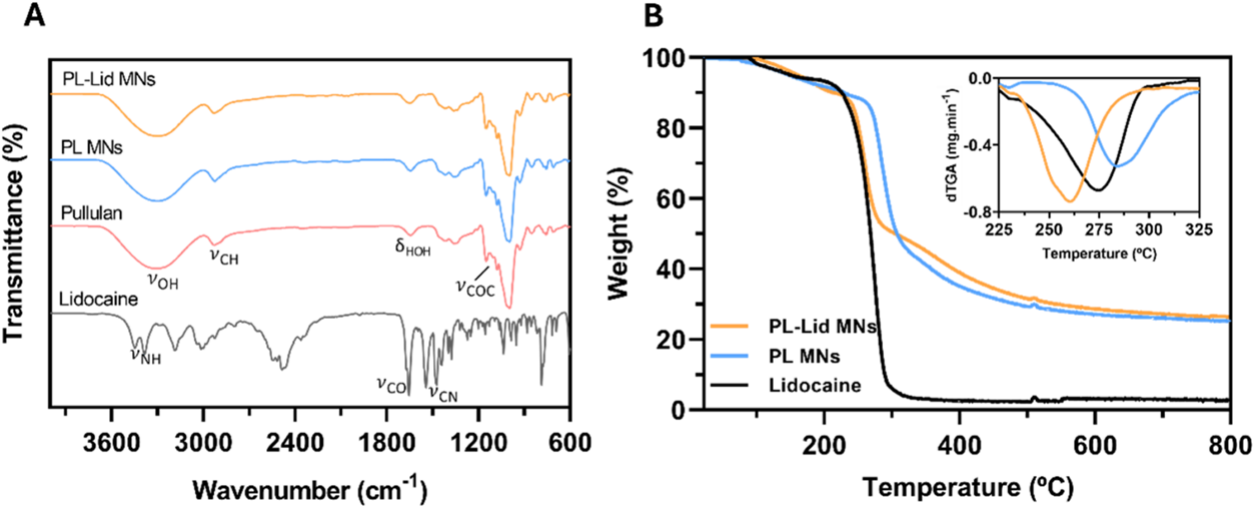
FTIR-ATR spectra of PL-Lid MNs, PL MNs, and the individual components (pullulan and lidocaine) (A); thermograms of PL-Lid MNs, PL MNs, and Lid, together with the inset displaying the derivative plot of the respective samples (B).

Regarding the spectra of lidocaine, its characteristic peaks are associated with the N–H stretching vibration (ν_NH_) observed at 3451 and 3385 cm^–1^, as well as the strong peak at 1655 cm^–1^ owing to the C = O stretching vibration (ν_CO_).^53^ The two sharp bands visible between 1450–1550 cm^–1^ are attributed to C–N stretching (ν_CN_).^46,54^ The spectrum of PL-Lid MNs does not show evidently these characteristic peaks of lidocaine, certainly due to its lower concentration in the formulation (10% w/w). Moreover, no significant shifts in the characteristic peaks of PL were perceived when compared with the previous spectra of PL and PL MNs, suggesting that there are no significant chemical interactions between pullulan and lidocaine.^55^

### 3.3 Thermogravimetric Analysis (TGA)

Thermal stability is a determinant factor when it comes to the fabrication of biomedical devices. Many procedures require thorough sterilization of the materials before usage, often conducted by heat.^56^ The thermograms of PL and PL-Lid MNs and lidocaine are shown in Figure 3B. The common weight loss visible up to 100 °C in thermograms of polysaccharides, due to water evaporation, is absent in the PL and PL MNs samples, since the MNs were dried after fabrication, and pullulan is nonhygroscopic.^57^ The thermogram of the PL MNs is consistent with the typical thermal behavior of pullulan, displaying a single-step weight loss between 250 and 300 °C, a maximum decomposition temperature (T_dmax_) of 284 °C, and a final residue of 25%.^58–60^ The thermogram of lidocaine is also in line with other studies, displaying a single-step weight loss with 55% weight residue at ∼250 °C, a T_dmax_ of 275 °C, and a final mass residue of 2%.^61^ Regarding the thermogram of PL-Lid MNs, a single-step weight loss is also observed, however, exhibiting a lower T_dmax_ of 260 °C in comparison to its main components, and a final mass residue of 27%.^62^ Although lidocaine is a minor component of the microneedles, its incorporation in the pullulan matrix certainly weakens the interactions between the polymeric chains, owing to its aromatic base structure, leading to an overall decrease in thermal stability. However, these findings ensure that for future clinical application of these MNs, all of the components can endure temperatures up to 200–220 °C without significant degradation and thus are deemed suitable for sterilization procedures that enable clinical translation.

### 3.4 Mechanical Performance

Mechanical strength is another key property regarding MNs, as it is essential for validating their skin penetration ability and avoiding failure of the tips upon insertion. For this, axial compression tests (Figure 4A) were performed, as they mimic the stress that MNs face upon skin insertion.^63^ The obtained force–displacement plots are shown in Figure 4B and reveal a continuous deformation of the MN tips over time for both PL MNs and PL-Lid MNs, with no fracture points being observed. This behavior is in line with previous studies using pullulan MNs,^35,36^ as well as other biobased polymers, such as carboxymethyl cellulose,^64^ and chitosan.^65^ The produced PL and PL-Lid MNs are able to sustain up to 1.6 ± 0.1 and 1.5 ± 0.2 N needle^–1^, respectively, without reaching failure. The obtained values considerably surpass the 0.1 N needle^–1^ threshold reported for proper skin insertion,^2,18,66^ and the mechanical performance of almost all dissolvable MNs for lidocaine administration reported so far, including several multicomponent,^67,68^ cross-linked,^4,26^ and layered MNs,^69^ displaying, overall, adequate mechanical strength for skin insertion. A reduction in the overall mechanical strength can be observed for PL-Lid MNs comparatively to PL MNs, which is in accordance with trends observed in other studies after drug loading.^4,36,64^ This effect can be discussed based on the increased free volume between pullulan’s chains as a consequence of lidocaine incorporation in its matrix, decreasing to some extent its intermolecular forces, and by extension, exhibiting a slightly lower mechanical performance.^70^

**Figure 4:**
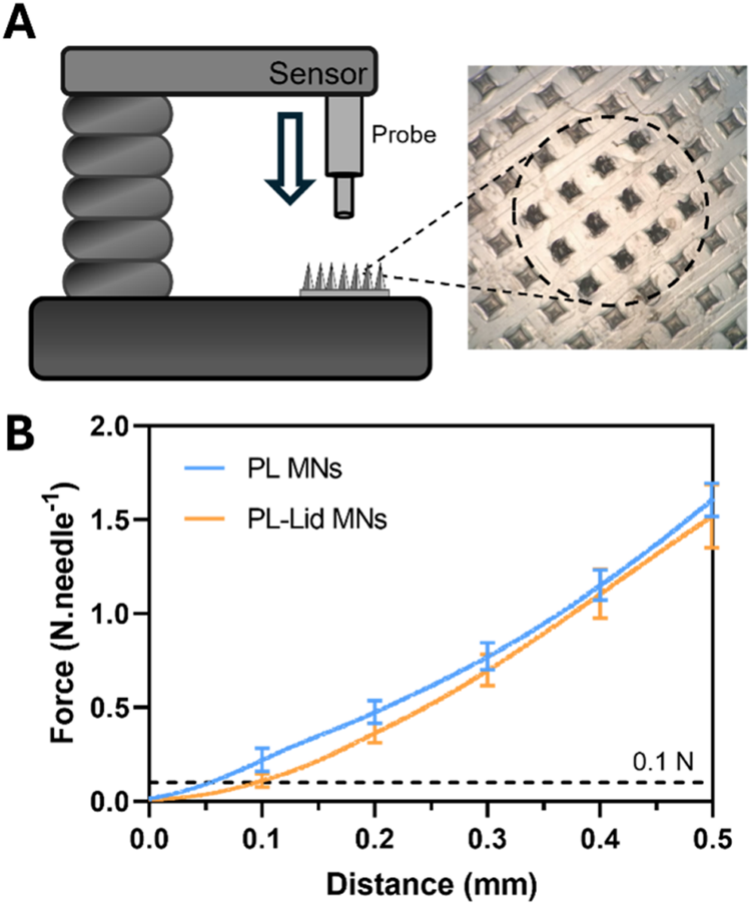
Visual representation of the axial compression test and optical micrograph of the bent needles after being subjected to force (A); and force–displacement curves for PL MNs and PL-Lid MNs patches (B).

### 3.5 Insertion Tests in a Parafilm M Model

To study the skin insertion potential of the developed MN patches, tests on a Parafilm M model (Figure 5A), which is a quick, easy-to-handle, and reliable approach for attaining preliminary data on the skin penetration profile of MNs were conducted.^71^ As shown in Figure 5B, both PL and PL-Lid MNs showed excellent penetration capability through the first 3 layers of the Parafilm M model but barely reached the fourth layer. Specifically, both patches were able to achieve 100% and over 98% insertion up to the first and second layers, respectively. At the third layer, PL MNs showed 93% insertion, while PL-Lid MNs displayed 95%. These values are validated by the micrographs exposed in Figure 5C, where the perforation marks indicate successful penetration of the first, second, and third layers of Parafilm M, which correspond to depths of 127, 254, and 381 μm, respectively. The soft marks on the fourth layer indicate that the tip was able to scratch but not pierce this layer, which is an expected result since the depth of this layer (508 μm) surpasses the average height of the needles. Thus, these preliminary insertion results demonstrate the good penetration ability for PL and PL-Lid MNs, with the incorporation of lidocaine not influencing this property, and corroborate the mechanical strength and sharpness results described above for the obtained needles. Moreover, the performance of these MNs exceeds in a great extent that of other lidocaine-loaded MNs, for instance, lidocaine-loaded gelatin methacrylate MNs, which only reached the second Parafilm M layer corresponding to a depth of roughly 200 μm, for a needle height of 600 μm.^21^

**Figure 5:**
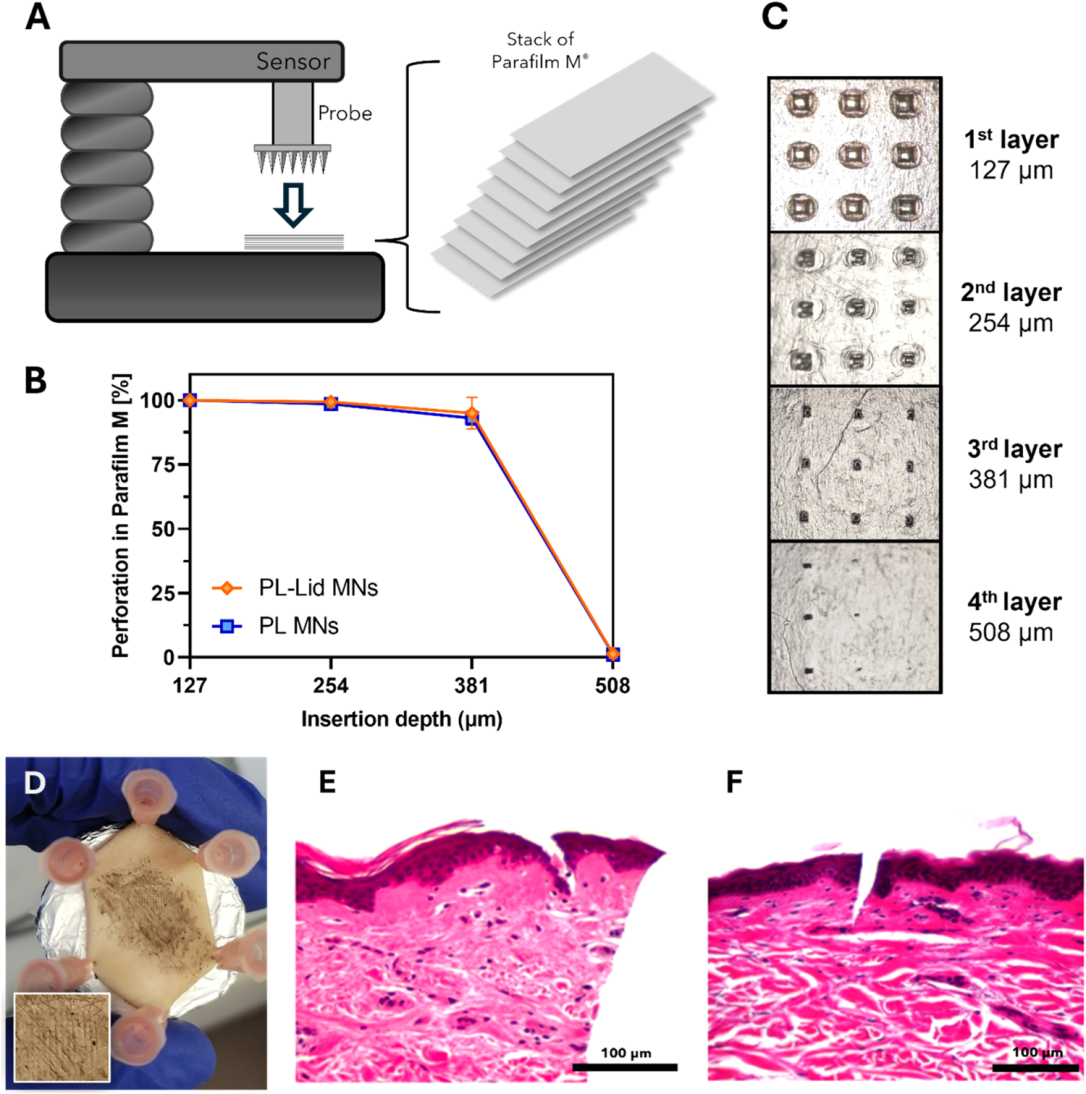
Schematic representation of the MN insertion test using a stack 8 layers of Parafilm M (A); percentage of MN perforations per layer of Parafilm M (B); photographs of insertion marks in each parafilm M layer (C); photograph of *ex vivo* human abdominal skin tissue after MNs insertion, fixed by hypodermal needles and stained with China ink (D); close-up of the patch insertion marks (inset); and histological cross sections of abdominal skin tissue pierced by PL-Lid MNs, hematoxylin, and eosin stain (E, F).

### 3.6 *In Vitro* Insertion in Excised Human Skin

To validate the skin insertion capability of the produced PL-Lid MNs, histological analysis of *ex vivo* human abdominal skin was performed after application of the MN patches using a constant force of 40 N during 30 s. Figure 5D shows the excised skin after PL-Lid MN insertion, with the inset displaying spaced insertion marks, evidenced by the China ink staining. In Figure 5E,F, a clear penetration of both the SC and the epidermis is observed, with the insertions successfully reaching the superficial dermis. However, the observed MN penetration depth (ca. 90 μm) is significantly lower than the actual height of the produced MNs (485 μm), corresponding to about 19% of the total height. Similar results were reported by Xie and colleagues for lidocaine-loaded sodium carboxymethyl cellulose MNs, where only 12.5% of the total height was able to be inserted in rat skin.^25^ However, other works have reported higher penetration depths for MNs intended for lidocaine administration, such as lidocaine-coated poly­(l-lactide) MNs that reached 200 μm for a total height of 400 μm.^72^ A possible explanation for this dissimilar behavior can be credited, on the one hand, to the high elasticity of human abdominal skin tissue,^73^ which is known to limit the penetration depth of MNs,^55,63^ and on the other hand, to high skin variability due to different donor types (rats, humans, pigs). Additionally, different polymers, concentrations, and molecular weights also have a relevant impact on the mechanical properties of the MNs. Nevertheless, the produced PL-Lid MNs were indeed able to overcome the barrier of the SC and epidermis, underlining their potential for local anesthesia application.

### 3.7 *In Vitro* Release Profile in PBS

The release profile of lidocaine from PL-Lid MNs in PBS is shown in Figure 6A. Overall, a quick zero-order release is observed over the first 10 min, steadily decreasing afterward due to depletion of lidocaine from the patches. Specifically, at 3, 5, and 10 min, the PL-Lid MNs patches were able to release 24, 42, and 79% of lidocaine, respectively, reaching a cumulative drug release of 92% at the end of 20 min. This release profile was fitted to the Korsmeyer–Peppas model, obtaining a diffusional constant (*n*) of 1.25 (*R* ^2^ = 0.9978). This suggests a super case II transport release mechanism, a typical behavior of hydrophilic materials, where pullulan is eroded over time as a consequence of its dissolution.^74^ These results showcase the swift dissolution capabilities of pullulan, which contribute to a very low onset time, while releasing relevant amounts of lidocaine for local anesthesia, higher than previous studies on this topic. For instance, Mao and colleagues developed dissolvable MNs made of polyvinylpyrrolidone (PVP), poly­(vinyl alcohol) (PVA), and sodium hyaluronate (HA) for lidocaine delivery, revealing 90% lidocaine release after 1 h, also in PBS.^18^ In another study, Xia et al.^4^ fabricated a bilayered MN system composed of HA, PVP, and a cross-linked network of chondroitin sulfate able to release approximately 60% of loaded lidocaine after 60 min. When considering clinical practices that require rapid anesthesia, the PL-Lid MNs developed in this study are deemed preferable due to their fast release.

**Figure 6:**
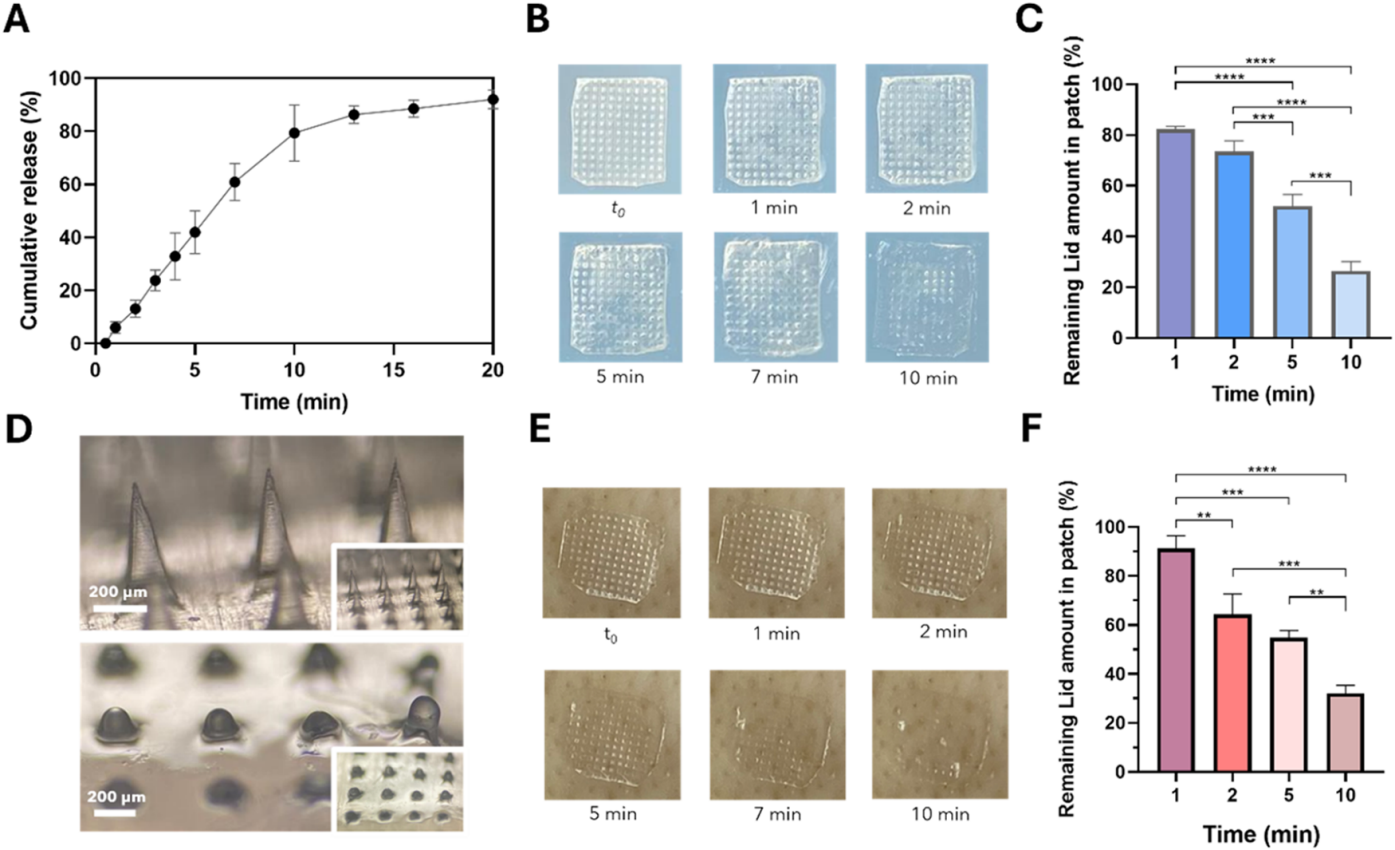
Lidocaine release profile in PBS at pH 7.4 and 37 °C (A); photographs of PL-Lid MNs before (*t*_0_) and after (1, 2, 5, 7, and 10 min) insertion in an agarose hydrogel covered with a parafilm M layer (B); amount of retained lidocaine in the patches after insertion in agarose hydrogel (C); optical micrographs of MNs before and after 5 min of insertion in agarose hydrogel (D); photographs of PL-Lid MNs before (*t*_0_) and after (1, 2, 5, 7, and 10 min) insertion in porcine ear skin (E); amount of retained lidocaine in the patches after insertion in porcine ear skin (F).

### 3.8 *In Vitro* Dissolution Assay in Agarose Hydrogel and Porcine Ear Skin

Given the importance of the dissolving character of these MNs, two different models were employed to validate this property, namely, an agarose hydrogel and porcine ear skin.^63^ Figure 6B reveals photographs of the progressive dissolution of a PL-Lid MN patch, where its structural integrity is lost over time, as observed by the gradual loss of definition of the tips. This confirms that, at 10 min, complete dissolution of the tips is achieved, as well as most of the base of the patch. The amount of lidocaine retained in the MNs patch after insertion in the agarose hydrogel model is presented in Figure 6C, showing that the patches retain 82, 74, 52, and 26% of lidocaine after 1, 2, 5, and 10 min of insertion into the agarose hydrogel, respectively. These data are comparable to those of the obtained release profile in PBS and show that these patches can undergo a gradual dissolution beginning with the inserted tips until reaching the base of the patch, as elucidated by the state of the tips presented in Figure 6D. These results are in accordance with the dissolution profile of the MNs in PBS. Similar findings were observed by Silva et al.,^64^ using diclofenac-loaded carboxymethyl cellulose MNs, which also resulted in the dissolution of the MNs tips after 10 min of insertion in an agarose gel.

The dissolution assay performed on porcine ear skin showed comparable results to those obtained in the agarose hydrogel, with a similar patch dissolution speed and loss of integrity over the same time points, as displayed in Figure 6E. Moreover, lidocaine quantification revealed that the patches retained 91, 64, 54, and 32% of lidocaine after 1, 2, 5, and 10 min of insertion into porcine skin, respectively (Figure 6F). Taken together, these results confirm the dissolution potential of PL-Lid MNs in two separate models, both exhibiting comparable behavior in terms of dissolution speed and retained lidocaine.

### 3.9 Cytocompatibility and Drug Release in Cell Cultures

Following the chemical and physical characterization of the developed MNs, their cytocompatibility toward skin cell lines was evaluated. Skin is a complex, multilayered organ that relies on consistent homeostasis to protect the body,^63^ accomplishing this role through a delicate balance and communication between different types of cells,^63^ namely, keratinocytes,^75,76^ fibroblasts,^77^ and macrophages.^78^ Therefore, the safety of the developed MNs was assessed toward HaCaT (keratinocytes), 3T3 (fibroblasts), and RAW 264.7 (macrophages) cell lines after 24 h exposure to PL MNs or PL-Lid MNs (assembly shown in Figure 7A). Of note, the full content of these MNs was dissolved in the cell culture medium to mimic their almost complete dissolution upon skin insertion. For each cell line, viability is expressed as percentages of controls cells’ viability (Figure 7B). The amounts of both pullulan and lidocaine used in the preparation of the MNs show no negative effect on the viability of these cells, which displayed values over 80% for the tested cell lines compared to their respective controls. In view of the guidelines of ISO 10993–5:2009­(E), a material is deemed to be cytotoxic if the cell viability decreases by 30% after exposure to a test agent. In addition, given that the biological half-life of lidocaine in the human body varies between 2 and 2.5 h,^3^ the obtained cell viabilities after 24 h of exposure validate the safety of the developed PL-Lid MNs for skin application.

**Figure 7:**
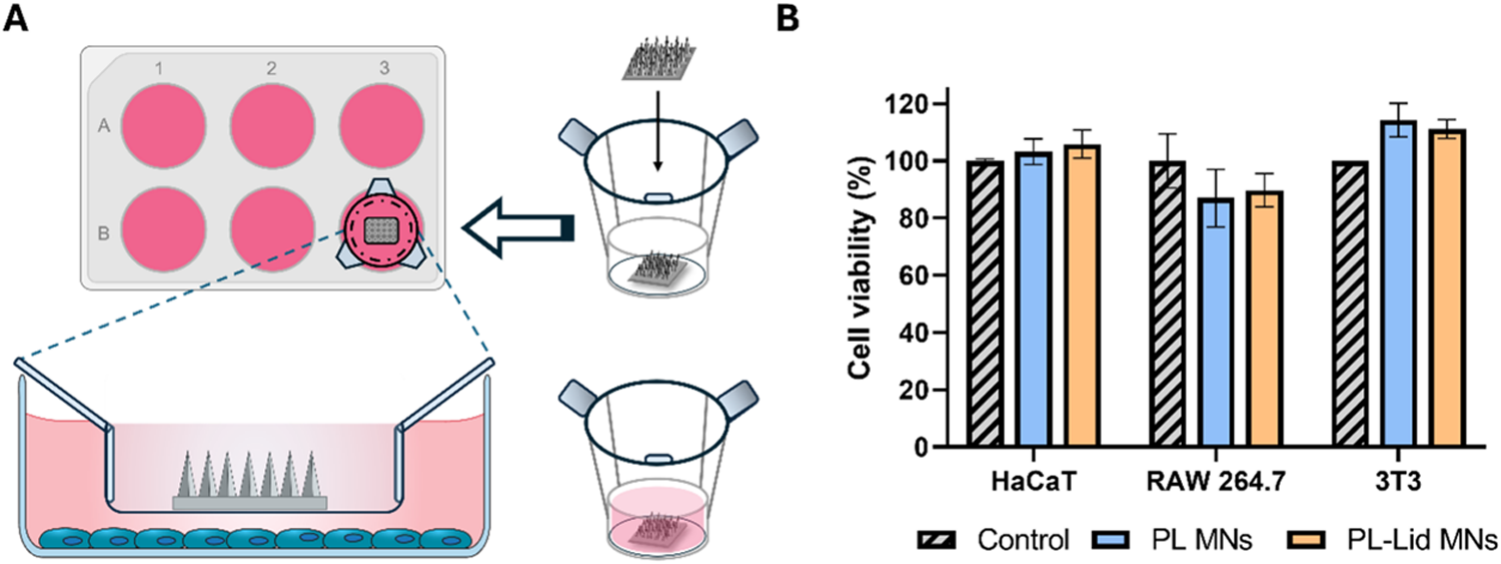
Experimental assembly used in cell viability assays (A). Cell viability (%) of HaCaT, 3T3, and RAW 264.7 cell lines after 24 h exposure to both PL MNs and PL-Lid MNs (B). Data are represented as mean ± standard deviation of 3 replicates. No statistically significant (*p* < 0.05) differences, relative to controls, were found.

The validation of the efficiency of MNs for drug application is often carried out following animal experimentation. However, guided by the 3Rs principle, the current regulatory framework strongly encourages the use of nonanimal alternatives, particularly *in vitro* tests, especially in the early stages of research (Directive 2010/63/EU of the European Parliament and the Council on animal experimentation). Thus, in this study, the efficacy of the PL-Lid MNs to deliver bioactive lidocaine was evaluated by using an *in vitro* model based on the F11 DRG neuronal cell line. However, it should be noted that to further validate this efficiency, *in vivo* tests should be considered in the future. In parallel to its known anesthetic effects, due to the blockage of sodium ion channels,^6^ lidocaine also activates TRPA1 and TRPV1 ion channels, provoking intracellular uptake of Ca^2+^ ions and endocytosis, in a dose-dependent manner.^42–44^ This induced endocytosis is followed by a compensatory exocytosis.^79,80^ The bioactivity of the lidocaine released from the MNs on neuronal TRP channels and, hence, on endocytosis–exocytosis was monitored through the use of a FM1–43 styryl dye. This nonpermeable dye reversibly stains the cell membranes, displaying a high quantum yield when bound to lipid membranes, while it is almost nonfluorescent when unbound in the extracellular medium (Figure 8A).^80,81^ Exposure to a depolarizing or excitatory agent affecting, *e*.*g*., intracellular calcium concentration, promotes rapid endocytosis and subsequential exocytosis in stained cells, leading to probe unload and loss of fluorescence, as schematized in Figure 8A.^80,82^ Figure 8B shows a dose-dependent significant decrease in the FM1–43 mean fluorescence intensity in cells fixed upon 5 min of exposure to control solutions of lidocaine concentrations of 7 mM (decrease of 18%) and 14 mM (reduction of 26%), with cells exposed to dissolved PL-Lid MNs behaving similarly to the 14 mM lidocaine concentration (reduction of 29%). Indeed, the lidocaine released after 5 min from the PL-Lid MN produces a similar effect as the *in situ* direct application of the 14 mM lidocaine solution to these neurons. The kinetics of fluorescence decrease can be observed in unfixed living cells, in live cell imaging assays using confocal microscopy (Figure 8C). Basal decay shows a gradual loss of fluorescence due to normal cell metabolic activity,^83^ where fluorescence intensity slowly decreases by 26% over 590 s with no sudden shifts. When a 14 mM lidocaine solution is directly added to these cells, a small fast increase (initial endocytosis), followed by an abrupt drop in fluorescence (compensatory exocytosis), is observed at the moment of application (160 s), where the fluorescence intensity is reduced by 24 and 32% for the overall and region of interest (ROI) measurements, respectively, over the course of 100 s. A similar, but more pronounced, effect is visible regarding the fluorescence intensity plot after administering a fully dissolved PL-Lid MN patch, where the intensity drops by approximately 46% in both measurements, in an interval of just 38 s. These results show that the cells respond swiftly to lidocaine exposure, arising from the addition of a control solution or from the complete dissolution of a PL-Lid MN patch, displaying loss of fluorescence originating from the release of FM1–43 from the cell membrane into the extracellular medium, where it is nonfluorescent. This is in line with the results displayed in Figure 8B, which show that the observed loss of fluorescence is proportional to lidocaine concentration. Overall, these results indicate that the produced PL-Lid MNs are able to, upon dissolution, efficiently release enough bioactive lidocaine to provoke a response similar (and, to some extent, greater) than a direct application of a lidocaine solution of similar concentration (14 mM) to the same neuronal culture. While a similar approach has been reported by Pyle and colleagues to visualize the synaptic activity with this probe,^84^ this work provides an unconventional approach for gauging the effect of dissolvable drug delivery devices through FM1–43 staining and fluorescence intensity measurements that serve as a foothold for further studies.

**Figure 8:**
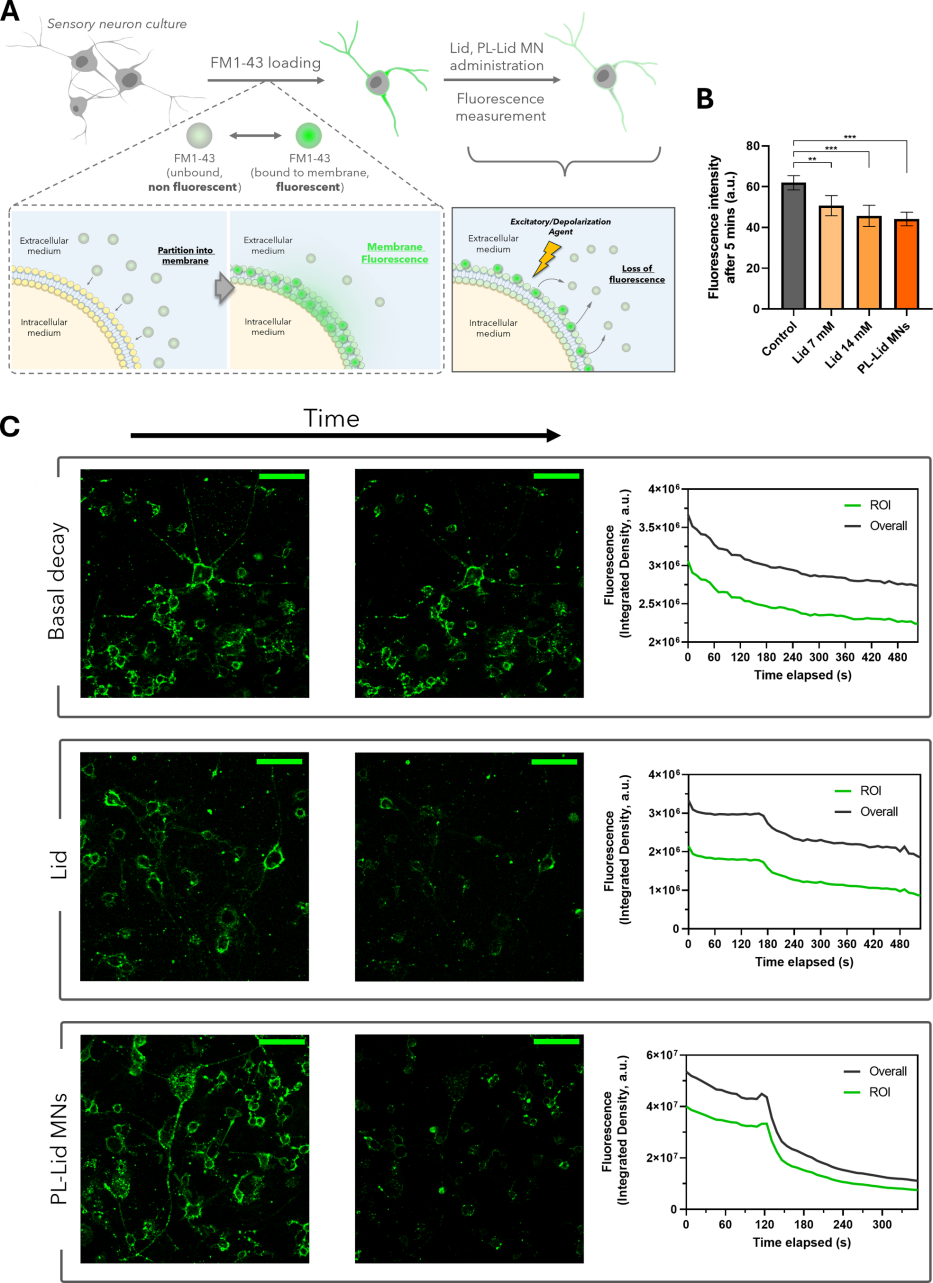
Schematic representation of the key mechanism of fluorescence loading and fluorescence decay after endocytosis and its compensatory exocytosis for indirect evaluation of lidocaine release (A). Graphic analysis of the mean fluorescence intensity of FM1–43-stained DRG neurons fixed after 5 min of exposure to Lid 7 mM, Lid 14 mM, and PL-Lid MNs (dissolving); unexposed cells were used as controls (B). Fluorescence intensity over time of neurons loaded with FM1–43 probe for three distinct conditions (unexposed control cells and cells exposed to Lid 14 mM or to a solution of dissolved PL-Lid MNs) (C): snapshots of the first and last frames *(left)*; plots of fluorescence intensity (au) over the full duration of the imaging recording *(right)*. Scale bar: 100 μm.

## 4 Conclusions

The present study aimed to assess the potential of pullulan for the fabrication of single-component dissolvable MNs for transdermal delivery of lidocaine. PL-Lid MNs were successfully produced through micromolding, yielding well-defined pyramidal MNs with an average height of 485 μm. These displayed thermal stability up to ca. 200–220 °C, adequate mechanical strength for skin insertion without tip breakage, and insertion potential to reach the dermis, as evidenced by the tests with excised human skin. *In vitro* drug release in PBS evidenced a fast release of lidocaine, reaching a cumulative release of 79% after 10 min, which was complemented by dissolution tests in an agarose gel and porcine ear skin, showcasing complete MN tip dissolution for the same duration. Additionally, safety evaluation against three distinct cell lines proved that the patches are safe for skin application. The efficacy of lidocaine release by the MNs was evaluated using an *in vitro* neuronal model through fluorescence intensity measurements based on the interaction between the FM1–43 probe, a DRG neuron cell line, and lidocaine. The results obtained demonstrated that the PL-Lid MNs have a similar effect on these neurons as a direct local application of 14 mM of lidocaine. In a nutshell, PL-Lid MNs owe their success to the outstanding properties of PL, which not only opens the way for scalable production from a sustainable source but also benefits from ease of handling, enabling future tailoring of this system according to patient needs for efficient pain control. Moreover, its swift dissolution ensures the delivery of significant lidocaine quantities with a low onset time, overall deeming PL-Lid MNs a valuable asset in the vast landscape of anesthetic delivery devices through minimally invasive devices, as well as in comparison with other MNs developed for this purpose, mostly prepared by combination of different polymers, and including synthetic ones that are typically less biocompatible and biodegradable.

## Supplementary Material







## Notes

Image stacks used in Figure 8C for fluorescence measurements are displayed in Supporting Information. Basal Decay (open with ImageJ) (PDF)
Lid (open with ImageJ) (PDF)
PL-Lid MNs (open with ImageJ) (PDF)
