# Supplementary figures and images for: Single Polysaccharide Dissolvable Microneedles for Painless Local Anesthesia: Fabrication, Characterization, and In Vitro Neuronal Imaging
Source: ACS Biomater Sci Eng. 2025 Aug 1;11(9):5426–39. doi: 10.1021/acsbiomaterials.5c00968 (PMC12818717; doi:10.1021/acsbiomaterials.5c00968)

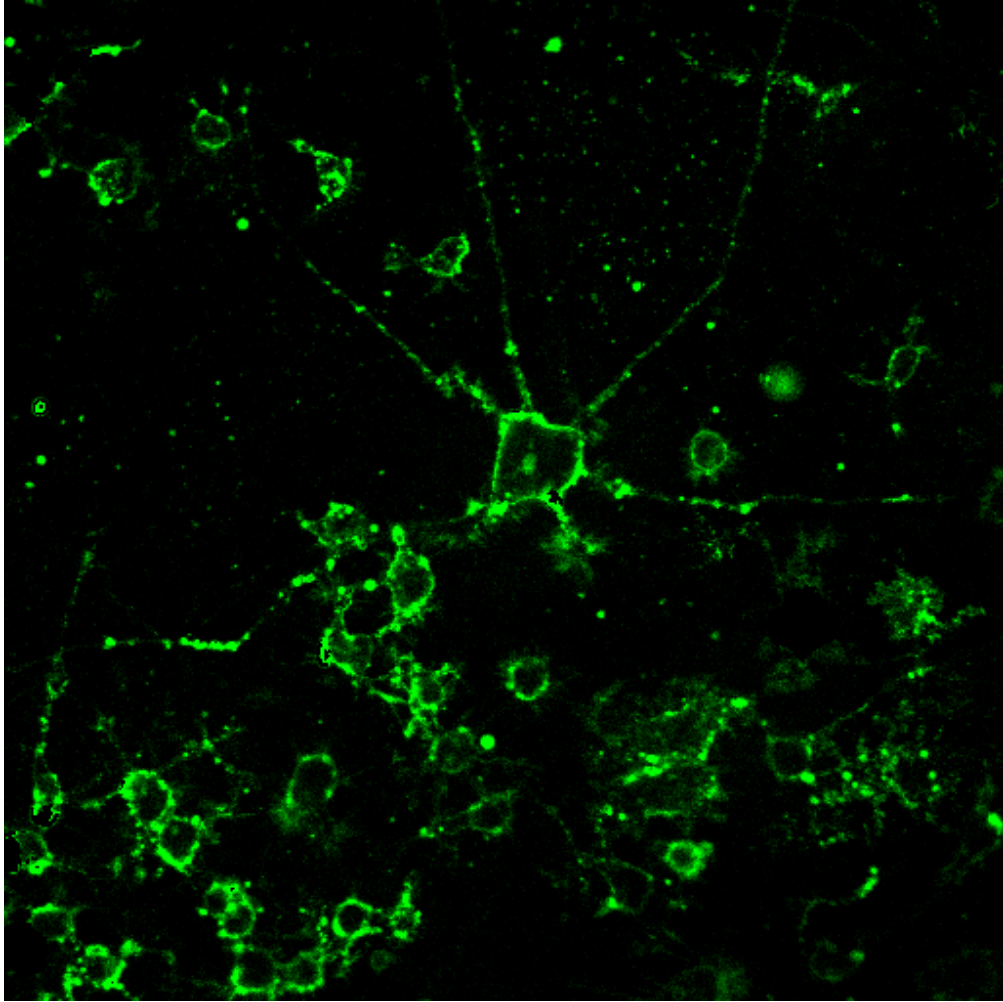

13004x13004mm (1 x 1 DPI)

Supplement: Supplementary file 1 [file ab5c00968_si_001.pdf]

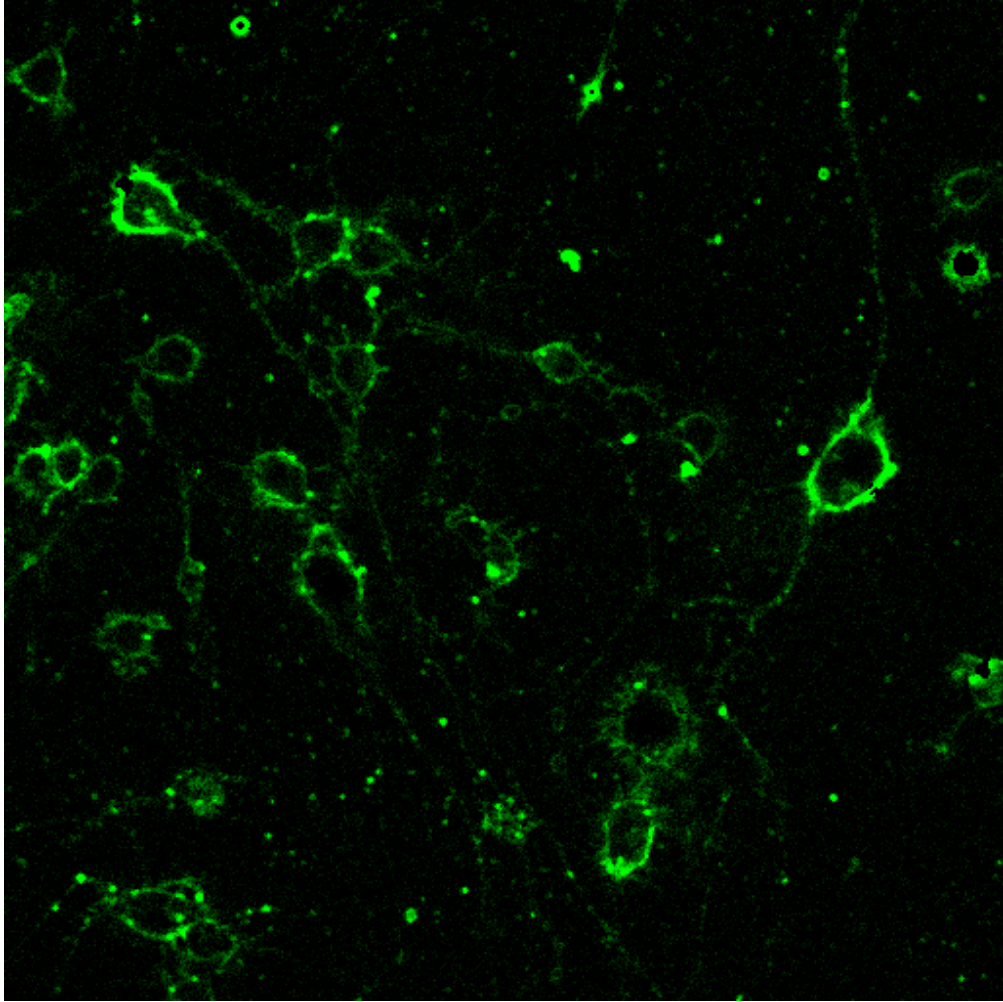

13004x13004mm (1 x 1 DPI)

Supplement: Supplementary file 2 [file ab5c00968_si_002.pdf]

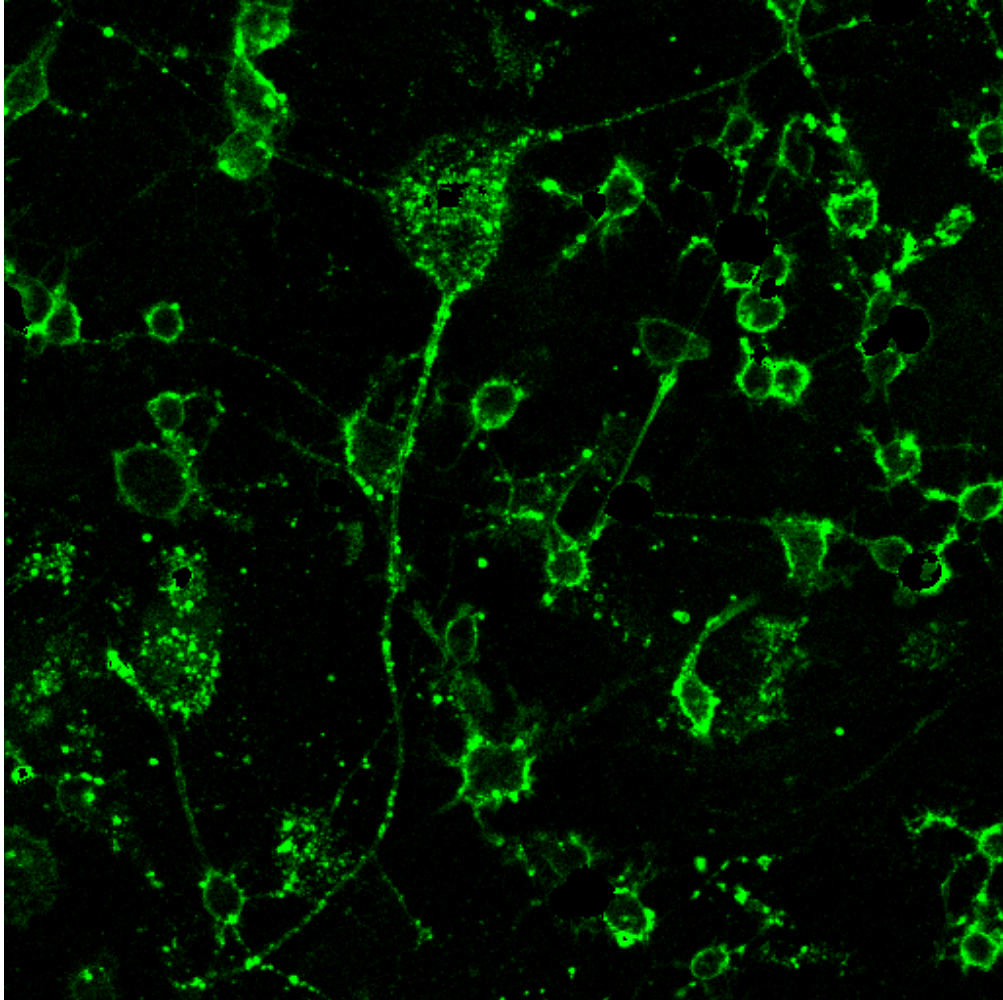

13004x13004mm (1 x 1 DPI)

Supplement: Supplementary file 3 [file ab5c00968_si_003.pdf]
